# Miscellaneous Items

**Published:** 1886-11

**Authors:** 


### MISCELLANEOUS ITEMS.

**"Are you** having much practice now?" asked an old doctor of a young beginner. "Yes, sir; a great deal, thank you." "Ah, I am glad to hear of it. In what line is your practice, particularly?" "Well, sir, particularly in economy."—*Reconstructives.*

**"Why will** you persist in drinking tea and coffee?" asked the doctor. 'A milk diet is the healthiest, it contains all the elements of the human, blood." "Very true," replied Boggs, swallowing a third cup of coffee; "but then, you know, I am not blood-thirsty."—*Med. World.*

**It is not** what people eat but what they digest that makes them strong. It is not what they gain but what they save that makes them rich. It is not what they read but what they remember that makes them learned. It is not what they profess but what they practice that makes them righteous.

**Marrying Drinking Men.**—Marrying men to reform them has never been a successful enterprise on the part of women. Girls are worth too much unmarried to sacrifice themselves to beat sense into the head of any man on God's footstool. Such a man does not wean so easily as a calf. He will go home only to sober up, and then not till the other places are closed. A girl will marry such a man, hoping that on next year he will be better; but the next year he will be worse. There are sober boys enough for all the girls; and there is no need for marrying a drunkard.—*New York Sun.*

**Young Wife.**—"There's a gentleman in the parlor, dear, who wishes to see you."

He—"Do you know who it is?"

Young Wife—"You must forgive me, my dear, but that cough of yours has worried me of late, and you take such poor care of your health, and—and O, if I were to lose you, my darling!" (Bursts into tears.)

He—"There, there, dear. Your fondness for me has inspired foolish and unnecessary fears. I'm all right; you mustn't be alarmed. But I'll see the physician, of course, just to satisfy you. Is it Dr. Pellett?"

Young Wife—"N-no, it is not a doctor; it's a—a—life insurance agent."—*Reconstructives.*

**"The Faith of a Little Child."**—At a certain country church it was decided by the members to assemble together at a given time to pray for rain, which was badly needed for the growing crops. At the appointed hour the people began to gather, and one littie fellow came trudging up with an umbrella almost as big as himself.

"What did you bring that for, youngster?" some one asked, with a smile.

"So's I wouldn't get wet going home," was the confident reply.

It is safe to say that no one in the large gathering had come similarly provided, and that not a house-wife of them all had set out her tubs and pans to catch the rain-water as it would pour from the roof in answer to the ascending petition.—*Pittsburg Post.*

**Rules For Living Well.**—The *Caterer*, in an article on "Good Living" considered from a culinary rather than a moral point of view, sums up the matter in the following sensible way:

If living well consists in living neither luxuriously nor expensively, in what, then, lies the secret? And how may the man of moderate means take advantage of it? The question is not a difficult one to answer. We will put the answer in the shape of a few short rules:

1. Buy with judgment.
2. Buy the best, for the best is always the cheapest.
3. Let your economy regulate the quantity, not the quality.
4. Let your cook be a cook, and one that knows how to utilize what is now thrown to the dogs, or otherwise wasted.
5. Study simplicity in the number of the dishes, and variety in the character of the meals.
6. Let the housewife be watchful enough to trace the leaks that are liable to spring in every larder.
7. Let the mistress be indeed the head of her own household and of her own kitchen.

